# In Older Patients Treated for Dizziness and Vertigo in Multimodal Rehabilitation Somatic Deficits Prevail While Anxiety Plays a Minor Role Compared to Young and Middle Aged Patients

**DOI:** 10.3389/fnagi.2018.00345

**Published:** 2018-10-30

**Authors:** Maren Dietzek, Sigrid Finn, Panagiota Karvouniari, Maja A. Zeller, Carsten M. Klingner, Orlando Guntinas-Lichius, Otto W. Witte, Hubertus Axer

**Affiliations:** ^1^Center for Vertigo and Dizziness, Hans-Berger Department of Neurology, Jena University Hospital, Jena, Germany; ^2^Biomagnetic Center, Jena University Hospital, Jena, Germany; ^3^Department of Otorhinolaryngology, Jena University Hospital, Jena, Germany

**Keywords:** vertigo, dizziness, multimodal treatment, interdisciplinary, day care, older age

## Abstract

**Objective:** Many patients with dizziness and vertigo are of older age. It is still unclear which age-associated factors play a role in the treatment of dizziness and vertigo. Therefore, age-associated characteristics of patients subjected to an interdisciplinary day care approach for chronic vertigo and dizziness were analyzed.

**Subjects and Methods:** 650 patients with chronic dizziness/vertigo subjected to a multimodal vestibular rehabilitation day care program were analyzed. Information concerning age, gender, medical diagnosis, medical consultations, technical diagnostics performed and therapy achieved before attending the clinic were collected. Furthermore, data were gathered using the Vertigo Severity Scale (VSS), Hospital Anxiety and Depression Scale (HADS), Mobility Inventory (MI), as well as the intensity of and the distress due to vertigo/dizziness using visual analog scales. As a follow-up, the VSS, HADS, MI, and the visual analog scales were collected again 6 months after attending the therapy program. Three age groups were compared to each other (<41, 41–65, and >65 years of age).

**Results:** One-third of the patients were older than 65 years. This group had typical diagnoses with mainly organic deficits. In contrast to the dominance of mainly multifactorial, organic deficits the older patients reported less medical consultations, fewer technical diagnostics and even fewer treatments than the younger patients. The elderly scored significantly lower in total VSS, in VSS-V (vestibular-balance subscale), in VSS-A (autonomic-anxiety subscale) and in HADS-anxiety. Psychological diagnoses were clearly associated to the younger patients. 424 patients (65.2%) completed the follow-up questionnaire 6 months after attending the therapy week. The older patients revealed improvements of VSS-V and the Avoidance Alone scale of MI as well as decreased distress due to vertigo/dizziness.

**Conclusion:** In the older patients, who took part in our vestibular rehabilitation program, mainly somatic deficits prevail while anxiety plays a minor role compared to young and middle aged patients. Older patients profited from vestibular rehabilitation especially in mobility and vestibular-balance. Therefore, vestibular rehabilitation programs for the elderly with a focus on physio- and occupational therapeutic interventions and less cognitive behavioral therapy may be reasonable.

## Introduction

Vertigo, dizziness and balance disorders are frequent complaints in the everyday medical practice. These symptoms cause significant restrictions on quality of life and the ability to work. In addition, they lead to a high risk for chronification (Yardley et al., 1998) and an increased risk of falls especially in the older population (Axer et al., 2010).

Lifetime prevalence of significant dizziness has been estimated to range between 17 and 30% and for vertigo between 3 and 10% (Murdin and Schilder, 2015). Inpatient treatment rates per year for acute dizziness were 55 per 100,000 and for peripheral vestibular disorder 41 of 100,000 (Renner et al., 2017). The 1-year prevalence of vestibular vertigo is 5%, and the 1-year incidence is 1.4 % (Neuhauser, 2016). However, a clear definition of when dizziness or vertigo becomes chronic is still lacking (Sloane et al., 2001). Chronic vertigo and dizziness may be defined as symptoms persisting longer than 1 (Brandt et al., 2010) or up to longer than 3 months (Staab, 2006). In addition, vertigo and dizziness may also be chronic if attacks often recur.

Especially in people of advanced age vertigo, dizziness and impaired balance are among the most common complaints (Aggarwal et al., 2000; Penger et al., 2017). They lead to less physical activity, worse functioning of the lower extremities and a higher risk of falls (Kollén et al., 2017). Thus, dizziness and vertigo contribute to a significant disability in older patients (Mueller et al., 2014) and have demonstrated to play a dominant role in determining the mobility, health, and quality of life in older patients. Therefore, developing an effective treatment of these impeding symptoms seems to be essential.

Positive long-term effects have been found following a thorough diagnostic process and specific treatment in a specialized tertiary care center. These effects proved to be persistent over a period of 2 years (Obermann et al., 2015). Risk factors for an unfavorable outcome were advanced age, severe disability, constant vertigo or dizziness, and concomitant back pain.

In addition, a considerable number of patients subjected to vestibular rehabilitation will be of older age and it is still unclear which age-associated factors may play a role in the treatment of dizziness and vertigo. Therefore, the intention of this study is the analysis of age-associated characteristics in the interdisciplinary day care approach for chronic vertigo and dizziness in our institution.

The Center for Vertigo and Dizziness of Jena University Hospital is a multidisciplinary outpatient clinic for patients with chronic vertigo, dizziness, or dysbalance. The interdisciplinary team consists of specialists in neurology, otolaryngology, and physical medicine and rehabilitation (PMR) as well as psychologists. Every patient subjected to the Center initially gets an individual diagnostic workup. The first evaluation is based on anamnesis, clinical examination, and available diagnostic findings. Further technical diagnostics can be performed such as advanced clinical neurophysiology, vestibular diagnostics as well as imaging procedures such as CT or MRI – if considered to be necessary. In addition, every patient receives a psychological assessment. Consecutively, every patient receives a medical diagnosis, a therapeutic conception and is evaluated if he may be suited for a 5-days multimodal day care treatment.

Here, we prospectively investigated all patients with chronic vertigo and dizziness subjected to this 5-days multimodal and interdisciplinary day care treatment.

## Materials and Methods

### Patients

We prospectively analyzed patients with chronic dizziness and vertigo subjected to a day care multimodal treatment program in the Center for Vertigo and Dizziness of Jena University Hospital between June 2013 and March 2017. Dizziness and vertigo were defined to be chronic if symptoms persisted at least for 3 months or attacks recurred often in the last 3 months (≥5 days with symptoms/month). This study was carried out in accordance with the recommendations of ICH harmonized tripartite guideline for Good Clinical Practice, as well as the Declaration of Helsinki with written informed consent from all subjects. The study was approved by the local ethics committee (number 5426-02/18).

The multimodal and interdisciplinary day care treatment took place from Monday to Friday with an average of 7 h of therapy per day. The elements of the multimodal group therapy were specific physiotherapeutic training, psychological assessment and CBT-based psychoeducation and group therapy, training of Jacobson’s muscle relaxation technique, health education, as well as medical evaluation and treatment. The group sizes varied between 8 and 10 patients.

### Assessment and Scores

At the first day the following data were collected: age, gender, and medical diagnoses. Patients were asked to describe their medical consultations, technical diagnostics performed and therapy achieved before attending the vertigo center.

Every patient got a medical diagnosis as defined at our center as well as a psychological evaluation. Major medical diagnoses were differentiated as psychological or somatic. The somatic diagnoses were classified into benign paroxysmal positional vertigo, vestibular neuritis, bilateral vestibulopathy, Meniere’s disease, vestibular schwannoma, vestibular paroxysmia, central vertigo, vestibular migraine, or multisensory deficit.

The major psychological diagnoses were distinguished as phobic postural vertigo, secondary somatoform, or somatoform (or functional). Vertigo/dizziness was classified as unspecific if the symptoms could neither be assigned to psychological nor to somatic etiology.

However, as these psychological diagnoses are not generally used outside Germany, the definition of these entities demand further explanation. Definitions and criteria used for chronic conditions of dizziness/vertigo are collected in Table 1. Recently, criteria for persistent postural-perceptual dizziness (PPPD) were defined by an expert panel (Staab et al., 2017). The concept of PPPD is independent of a specified underlying pathophysiologic process and may be present alone or co-exist with other conditions. Our patients with psychological diagnoses as well as the patients classified as unspecific can also be subsumed under the diagnosis PPPD. In contrast, we intentionally chose the classification into phobic postural vertigo, somatoform, and secondary somatoform as these classifications inherently assign these patients to major psychological mechanisms responsible for the perpetuation of dizziness in these cases.

**Table 1:** Definitions of “non-organic” dizziness/vertigo.

Diagnosis	Symptoms/criteria
Persistent postural-perceptual dizziness (PPPD) (Staab et al., 2017) (Dieterich and Staab, 2017)	(A) One or more symptoms of dizziness, unsteadiness, or non-spinning vertigo are present on most days for 3 months and more. (1) Symptoms are persistent, but wax and wane. (2) Symptoms tend to increase as the day processes but may not be active throughout the entire day. (3) Momentary flares may occur spontaneously or with sudden movements. (B) Symptoms are present without specific provocation but are exacerbated by (1) Upright posture. (2) Active or passive motion without regard to direction or position. (3) Exposure to moving visual stimuli or complex visual patterns. (C) The disorder usually begins shortly after an event that causes acute vestibular symptoms or problems with balance, though less commonly, it develops slowly. (1) Precipitating events include acute, episodic, or chronic vestibular syndromes, other neurologic or medical illnesses, and psychological distress. (a) When triggered by an acute or episodic precipitant, symptoms typically settle into the pattern of criterion A as the precipitant resolves, (b) But may occur intermittently at first, and then consolidate into a persistent course. (c) When triggered by a chronic precipitant, symptoms may develop slowly and worsen gradually. (D) Symptoms cause significant distress or functional impairment. (E) Symptoms are not better attributed to another disease or disorder
Phobic postural vertigo (Dieterich et al., 2016) (Brandt, 1996)	(1) Postural dizziness and subjective stance and gait unsteadiness without. this being evident to an observer; normal findings in neuro-otologic tests. (2) Light-headedness with varying degrees of unsteadiness of stance and gait, attack-like fear of falling without actually falling, in part also unintentional body swaying of short duration. (3) Typical situations known to be external triggers of other phobic syndromes (e.g., bridges, driving a car, empty rooms, long corridors, large crowds of people in a store, or restaurant) or during visual stimulation (e.g., cinema, television, and store). (4) Symptoms improve or resolve during sporting activities and during more complicated balance conditions, whereas they reappear at rest or under simpler conditions (e.g., standing after cycling). (5) Generalization of the symptoms and increasing avoidance of triggering stimuli. vegetative disturbances and anxiety. (6) Symptoms improve after imbibing a little alcohol. (7) Initially there is often a structural vestibular illness or special psychosocial stress situations. (8) Obsessive-compulsive and perfectionistic personality traits and reactive-depressive symptoms during the course of the disease.
Somatoform (Feuerecker et al., 2015) (Eckhardt-Henn and Dieterich, 2005)	The diagnosis of somatoform dizziness is derived from the diagnostic criteria for Somatic Symptom Disorder noted in DSM 5 (Frances, 2013): (A) One or more somatic symptoms that are distressing or result in significant disruption of daily life. (B) Excessive thoughts, feelings, or behaviors related to the somatic symptoms or associated health concerns as manifested by at least one of the following: (1) Disproportionate and persistent thoughts about the seriousness of one’s symptoms. (2) Persistently high level of anxiety about health or symptoms. (3) Excessive time and energy devoted to these symptoms or health concerns. (C) Although any one somatic symptom may not be continuously present, the state of being symptomatic is persistent (typically more than 6 months).
Secondary somatoform (Feuerecker et al., 2015) (Eckhardt-Henn and Dieterich, 2005)	Secondary somatoform dizziness is initiated by an initial (but reversible) organic deficit causing dizziness or vertigo. The organic dysfunction often resolves (e.g., vestibular neuritis or benign paroxysmal positional vertigo that resolves) but a (secondary) somatoform dizziness develops in follow-up.
Functional dizziness (Brandt et al., 2015)	(1) Chronic spontaneous dizziness or unsteadiness lasting for months or longer. (2) Dissociation between objective balance tests and subjective imbalance. (3) Fear of falls without a history of falls. (4) Improvement during bodily activity, mental distraction or after alcohol consumption. (5) Inappropriate excessive anxiety or fear of impending doom. (6) Dizziness combined with non-vestibular or non-balance symptoms. (7) Situational or social events as triggers of dizziness and avoidance behavior. (8) Rotational vertigo without concurrent spontaneous nystagmus. (9) Unusual or bizarre postural and gait patterns. (10) Chronic unsteadiness and dizziness following transportation in vehicles.


In addition, different scores were collected: The Vertigo Severity Scale (VSS) is an assessment tool to quantify vertigo and dizziness symptoms (Yardley et al., 1992). It consists of two subscales, the VSS-V for vestibular-balance and VSS-A for autonomic-anxiety (Kondo et al., 2015).

The Hospital Anxiety and Depression Scale (HADS) is a self-report instrument for screening for anxiety and depression in medical outpatient settings (Andersson, 1993). It has been shown to also be a helpful screening for general psychological distress in dizzy patients (Piker et al., 2015).

The Mobility Inventory (MI) (Chambless et al., 1985) measures the patients’ avoidance behavior in various, mostly agoraphobic situations both when they are accompanied by someone (Avoidance Accompanied scale) and when they are alone (Avoidance Alone scale) (Chambless et al., 2011).

In addition, intensity of vertigo/dizziness and the distress due to vertigo/dizziness were quantified using a visual analog scale from 0 to 10.

Six months after attendance of the day care therapy program, patients were contacted via mail and asked to fill out a questionnaire consisting of the VSS, HADS, MI, and the visual analog scales for intensity of vertigo/dizziness and distress due to vertigo/dizziness.

### Statistics

IBM SPSS Statistics version 19 was used for statistical analysis of the data. In order to analyze age dependent differences three groups were built: patients younger than 41 years, patients between 41 and 65 years, and patients older than 65 years of age.

Descriptive statistics were used to characterize demographic data and baseline characteristics of the patients. Pearson Chi square test was used to detect differences between the age groups in baseline characteristics of patients, their medical consultations, technical diagnostics and therapy before attending the vertigo center. One-way ANOVA was used to detect differences in the scores between the age groups.

The unpaired *t*-test was used to compare VSS, HADS, and MI between individual groups. The paired *t*-test was used to compare the scores of the baseline assessment and the 6-months-follow-up. Generally, a two-sided significance level of 1% was assumed as multiple tests were performed.

## Results

650 patients were enrolled in this study with 105 patients younger than 41 years (group 1), 326 patients between 41 and 65 years (group 2), and 219 patients older than 65 years (group 3). 61% were female and 39% male. The gender ratio did not differ between the three age groups (Table 2).

**Table 2:** Baseline characteristics of patients, their medical consultations, technical diagnostics and therapy before attending the vertigo center.

	All patients (*n* = 650)	Age group < 41 (*n* = 105)	Age group 41–65 (*n* = 326)	Age group > 65 (*n* = 219)	
					
	% (*n*)	% (*n*)	% (*n*)	% (*n*)	Pearson Chi square
Gender	f: 61%, m: 39%	f: 60%, m: 40%	f: 61%, m: 39%	f: 61%, m: 39%	2.037, *p* = 0.437
Permanent dizziness	53.2% (*n* = 346)	63.8% (*n* = 67)	48.5% (*n* = 158)	55.3% (*n* = 121)	7.032, *p* = 0.006
Attacks of vertigo/dizziness	52.9% (*n* = 344)	36.2% (*n* = 58)	62.0% (*n* = 202)	38.4% (*n* = 84)	36.839, *p* < 0.001
Falls	29.7% (*n* = 193)	18.1% (*n* = 19)	31.3% (*n* = 102)	32.9% (*n* = 72)	88.62, *p* = 0.003
Medical consultation in the last year	86% (*n* = 559)	93.3% (*n* = 98)	88.3% (*n* = 288)	79.0% (*n* = 173)	10.951, *p* = 0.002
General practioner	69.2% (*n* = 450)	80.0% (*n* = 84)	71.8% (*n* = 234)	60.3% (*n* = 132)	
Otolaryngologist	71.8% (*n* = 467)	81.0% (*n* = 85)	76.4% (*n* = 249)	60.7% (*n* = 133)	
Orthopedist	41.5% (*n* = 270)	48.6% (*n* = 51)	45.7% (*n* = 149)	32.0% (*n* = 70)	
Neurologist	59.8% (*n* = 360)	60.0% (*n* = 63)	58.3% (*n* = 190)	48.9% (*n* = 107)	
Psychiatrist	6.8% (*n* = 44)	7.6% (*n* = 8)	9.8% (*n* = 32)	1.8% (*n* = 4)	
Psychotherapist	12.8% (*n* = 83)	20.0% (*n* = 21)	15.0% (*n* = 49)	5.9% (*n* = 13)	
Emergency department	32.9% (*n* = 214)	44.8% (*n* = 47)	33.7% (*n* = 110)	26.0% (*n* = 57)	
Non-medical practioner	18.2% (*n* = 118)	27.8% (*n* = 29)	20.2% (*n* = 66)	10.5% (*n* = 23)	
Technical diagnostics	80.2% (*n* = 512)	91.4% (*n* = 96)	81.0% (*n* = 264)	73.5% (*n* = 161)	8.902, *p* = 0.004
Brain MRI	77.1% (*n* = 501)	90.5% (*n* = 95)	78.2% (*n* = 255)	68.9% (*n* = 151)	
Cervical MRI	39.5% (*n* = 257)	90.5% (*n* = 41)	46.9% (*n* = 153)	28.8% (*n* = 63)	
CT	32.5% (*n* = 211)	30.5% (*n* = 32)	33.7% (*n* = 110)	31.5% (*n* = 69)	
X-ray	27.5% (*n* = 179)	31.4% (*n* = 33)	30.4% (*n* = 99)	21.5% (*n* = 47)	
ECG	44.6% (*n* = 290)	49.5% (*n* = 52)	45.7% (*n* = 149)	40.6% (*n* = 89)	
24-h ECG	35.2% (*n* = 229)	41.9% (*n* = 44)	35.0% (*n* = 114)	32.4% (*n* = 71)	
Echocardiography	17.5% (*n* = 114)	21.9% (*n* = 23)	17.8% (*n* = 58)	15.1% (*n* = 33)	
Cardiac catheter examination	6.0% (*n* = 39)	2.9% (*n* = 3)	5.5% (*n* = 18)	8.2% (*n* = 18)	
Duplex sonography of brain arteries	50.6% (*n* = 329)	43.8% (*n* = 46)	55.8% (*n* = 182)	46.1% (*n* = 101)	
Neurophysiologic testing	24.8% (*n* = 161)	33.3% (*n* = 35)	24.2% (*n* = 79)	21.5% (*n* = 47)	
Caloric tests	47.5% (*n* = 309)	50.5% (*n* = 53)	50.6% (*n* = 165)	41.6% (*n* = 91)	
Any kind of treatment	70.8% (*n* = 460)	77.1% (*n* = 81)	75.8% (*n* = 247)	60.3% (*n* = 132)	18.438, *p* < 0.001
Drugs	54.2% (*n* = 352)	55.2% (*n* = 58)	58.3% (*n* = 190)	47.5% (*n* = 105)	
Physiotherapy	44.9% (*n* = 292)	49.5% (*n* = 52)	49.1% (*n* = 160)	36.5% (*n* = 80)	
Psychotherapy	14.9% (*n* = 97)	19.0% (*n* = 20)	17.8% (*n* = 58)	8.7% (*n* = 19)	
Surgical intervention	2.8% (*n* = 18)	1.9% (*n* = 2)	3.1% (*n* = 10)	2.7% (*n* = 6)	


While attacks and permanent vertigo did not differ between the age groups, falls tend to increase over age (Table 2). Patients were asked to describe their medical consultations, technical diagnostics performed and their therapy before attending the vertigo center. With increasing age a decreasing number of patients had medical consultations, got technical diagnostics and received any kind of treatment.

**FIGURE 1:**
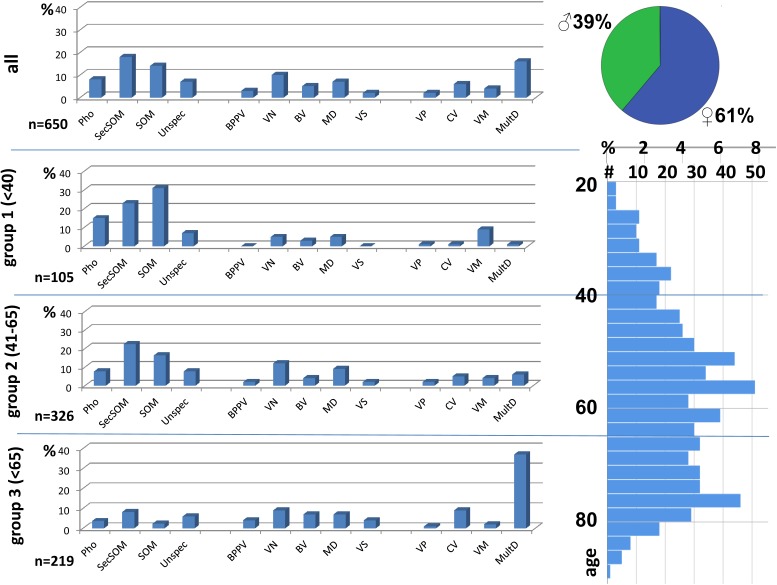
Diagnoses and age of patients with chronic vertigo or dizziness. Pho, phobic; SecSOM, secondary somatoform; SOM, somatoform; Unspec, unspecific; BPPV, benign paroxysmal positional vertigo; VN, vestibular neuritis; BV, bilateral vestibulopathy; MD, Meniere’s disease; VS, vestibular schwannoma; VP, vestibular paroxysmia; CV, central vertigo; VM, vestibular migraine; MultD, multisensory deficit.

Figure 1 shows the distribution of the different medical diagnoses over the age groups. A significant age-dependent decrease in the portion of psychological diagnoses was found while organic diagnoses in otorhinolaryngology or neurology increase with age. In particular, multisensory deficits and bilateral vestibulopathy are typical diagnoses in older age.

**FIGURE 2:**
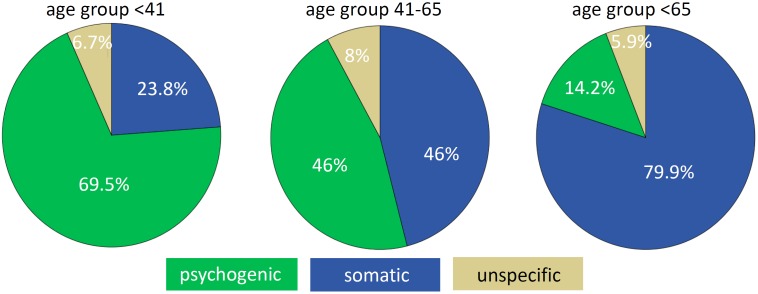
Classification of vertigo/dizziness according to somatic, psychogenic or unspecific (medically unexplained) origin in the three age groups.

Psychological diagnoses (such as somatoform, secondary somatoform and phobic dizziness) constituted 69.5% of diagnoses in the younger group and 46% in the middle-aged group, while in the older group only 14.2% of diagnoses are psychological (Figure 2).

**FIGURE 3:**
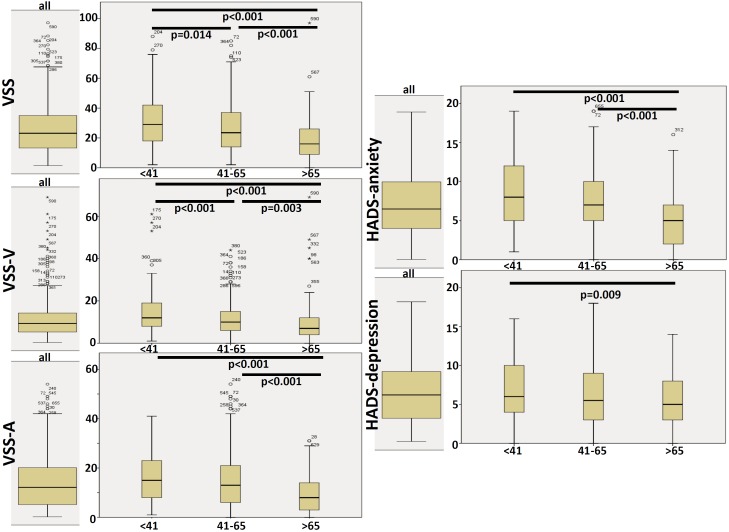
Box plots of VSS and HADS. Significant differences (student *T*-test) between age groups are shown at the top of the plots.

Table 3 shows a comparison of the Vertigo severity score (VSS), HADS, and MI between the age groups. No significant differences were found in MI. Figure 3 shows the box plots of VSS and HADS in the three age groups. VSS and VSS-V decreased over the age groups. VSS-A and HADS-anxiety were significantly lowest in the oldest age group compared to the younger groups.

**Table 3:** Comparison of vertigo severity score (VSS), hospital anxiety and depression scale (HADS), and mobility inventory (MI) between the age groups.

		Mean	Standard deviation	Standard error	One-way ANOVA	
					
					*F*	*P*
VSS total score	Age group 0–40	31.6	17.4	1.7	27.788	<0.001
	Age group 41–65	26.7	16.4	0.9		
	Age group 66–100	18.8	13.1	0.9		
	All	24.9	16.2	0.6		
Autonomic-anxiety (VSS-A)	Age group 0–40	16.6	10.0	1.0	26.960	<0.001
	Age group 41–65	15.3	11.0	0.6		
	Age group 66–100	9.6	7.9	0.5		
	All	13.6	10.3	0.4		
Vertigo-balance (VSS-V)	Age group 0–40	14.9	10.8	1.1	15.742	<0.001
	Age group 41–65	11.4	7.7	0.4		
	Age group 66–100	9.2	8.4	0.6		
	All	11.2	8.7	0.3		
HADS-anxiety	Age group 0–40	8.4	4.4	0.4	34.397	<0.001
	Age group 41–65	7.5	3.9	0.2		
	Age group 66–100	5.0	3.5	0.2		
	All	6.8	4.1	0.2		
HADS-depression	Age group 0–40	7.0	4.4	0.4	3.869	0.021
	Age group 41–65	6.2	4.0	0.2		
	Age group 66–100	5.6	3.7	0.3		
	All	6.1	4.0	0.2		
MI when accompanied	Age group 0–40	1.9	0.9	0.1	0.225	0.798
	Age group 41–65	2.0	1.1	0.1		
	Age group 66–100	2.0	1.0	0.1		
	All	2.0	1.1	0.1		
MI when alone	Age group 0–40	2.2	1.0	0.1	1.809	0.165
	Age group 41–65	2.3	1.2	0.1		
	Age group 66–100	2.4	1.3	0.1		
	All	2.3	1.2	0.1		


424 patients (65.2%) completed the follow-up questionnaire 6 months after attendance of the therapy week. Baseline comparison (unpaired *t*-test) between those patients who completed the follow-up questionnaires after 6 months to those who did not revealed no significant differences in baselines scores (VSS, HADS, or MI).

**FIGURE 4:**
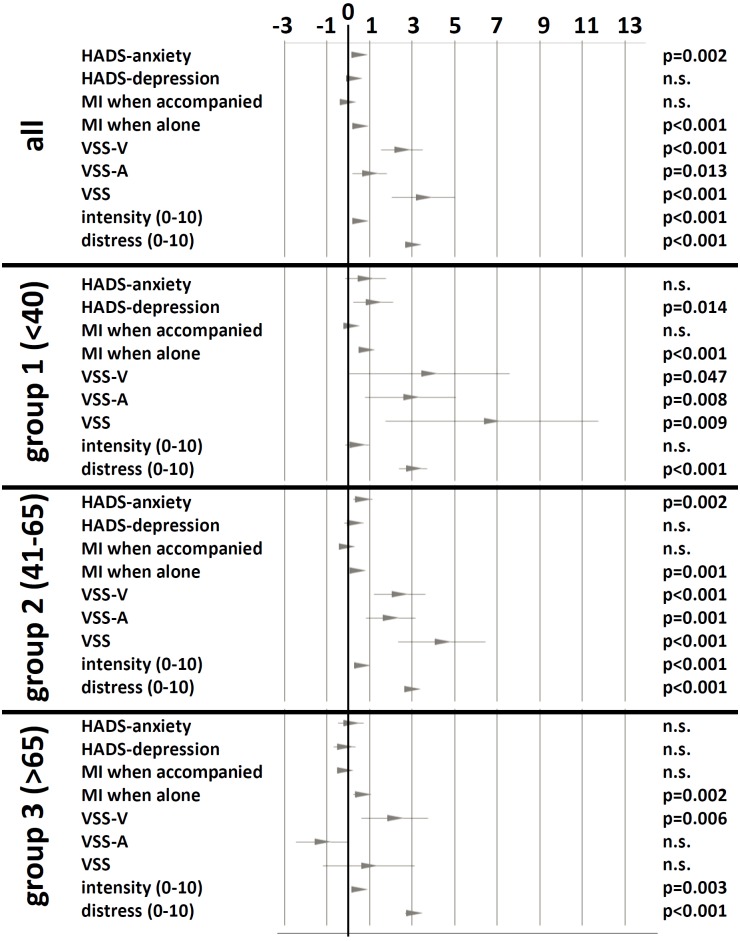
Change of scores before and 6 months after therapy week. The change is shown as 95% confidence interval and mean of differences. Significance levels of paired *T*-test are shown in the right column. n.s., not significant.

The change of the scores was analyzed according to the three age groups. Figure 4 shows the differences of scores before and 6 months after participation in the therapy week. All groups revealed improvements of VSS-V and mobility index when alone as well as decreased distress due to vertigo/dizziness.

## Discussion

There is increasing evidence that vestibular rehabilitation (Deveze et al., 2014) is effective in the recovery of balance function. This is especially true in the vestibular rehabilitation of the elderly (Mantello et al., 2008; Martins et al., 2016). In addition, the beneficial effect of a multimodal day care therapy was shown to last over at least 2 years (Obermann et al., 2015).

Although our data also demonstrated a persisting improvement over 6 months in patients with vertigo and dizziness after a 5 days outpatient multimodal rehabilitation program, the intention of this study focused on age-related aspects of chronic vertigo/dizziness.

Vertigo and dizziness are common symptoms spanning over all ages from adolescents (Langhagen et al., 2015) up to older ages. Here, vertigo and dizziness independently contribute to significant disability in patients older than 65 years (Mueller et al., 2014). In this population the 12-month prevalence of vertigo or dizziness ranges from 21% in those younger than 70 years to 37% in those older than 80 years (Mueller et al., 2014). Older persons with dizziness are less physically active and fall more frequently than people without dizziness (Kollén et al., 2017).

In our study about one third of patients were older than 65 years of age. Typical diagnoses of the older age comprised mainly degenerative somatic deficits such as multisensory deficits with gait disturbances, bilateral vestibulopathy, central vertigo (e.g., as consequence from stroke) and others. In contrast, psychological diagnoses such as somatoform or phobic postural vertigo/dizziness were clearly associated to the younger patients. It has to be pointed out that the patient population in this study does not result from a field study, but from a selected population referred to our center. It can be assumed that psychological processes may be significant factors for chronification of dizziness and vertigo (Tschan et al., 2011), so that the selection of patients for our vestibular rehabilitation program may probably increase the proportion of patients with behavioral complications.

In terms of disequilibrium, older patients are of particular importance (Kruschinski et al., 2010) as vertigo and dizziness increase as the load of somatic incapability increases over age. Therefore, disequilibrium in older people often is multifactorial in origin due to aging processes of vestibular and sensorimotor systems (Ishiyama, 2009) on the one hand and increasing load of different diseases and deficits over time on the other hand (Tuunainen et al., 2011).

In contrast to the dominance of mainly multifactorial, organic and degenerative reasons for vertigo/dizziness the older patients reported less medical consultations, fewer technical diagnostics and even fewer treatments than the younger patients. Moreover, it is remarkable that nearly all the younger patients had a cervical MRI before admission to the vertigo center, although no abnormality visible on MRI of the neck has been recognized as an explanation of vertigo and no convincing evidence of a cervical mechanism still exists (Brandt and Bronstein, 2001). This may emphasize the need for improvements in flows of diagnostic workup in primary care services not specialized on the treatment of dizzy patients (Grill et al., 2014).

The elderly scored significantly lower in total VSS, in VSS-V, in VSS-A as well as in HADS-anxiety than the younger patients. The elderly improved 6 months after therapy week especially in MI when alone and in VSS-V. That means, that the older patients have profited especially in mobility, autonomy and vestibular-balance. In contrast, anxiety-related scores did not reveal themselves to be as dominant as in the younger patients (shown in lower anxiety subscores of VSS and HADS as well) and, therefore, the older patients showed no significant changes in anxiety scores 6 months after therapy week.

The analysis of the major medical diagnosis of the patients is pragmatic in so far that it may stress the major proposed mechanism for dizziness and vertigo of the individual patient. The patients with psychogenic diagnosis – for instance – do not have any significant results in technical diagnostics and do not meet the diagnostic criteria for any organic diagnosis.

However, this necessarily is an oversimplification of the problem. The modern understanding on the interface between psychiatry and vestibular disease is that the psychiatric aspect is a comorbidity or a complication of a single or repetitive vestibular events. So, patients could well have two coexisting diagnoses, like unilateral vestibular deficit and a behavioral disorder.

**FIGURE 5:**
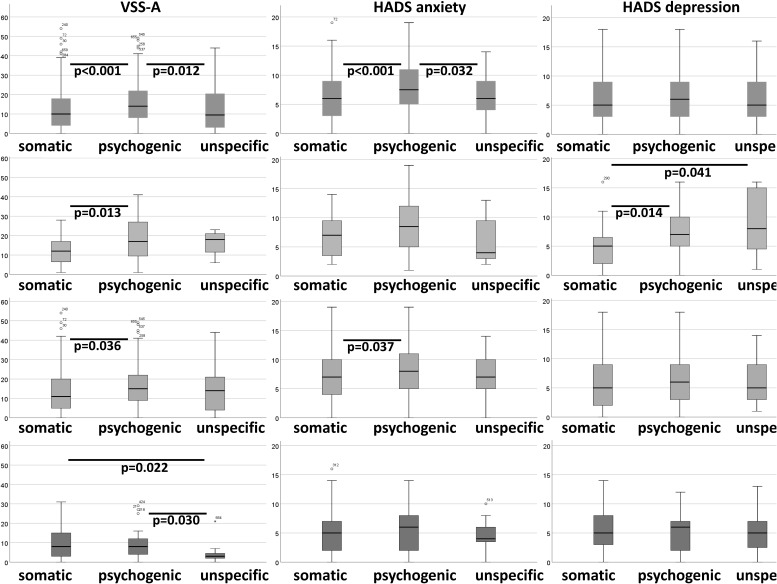
Boxplots of VSS-A, HADS anxiety, and HADS depression according to organic and non-organic diagnoses (psychogenic and unspecific).

Therefore, every patient in our center gets an organic and a psychological evaluation as well. As vertigo and dizziness are multidimensional conditions at least somatic and psychological assessments should be done in parallel. It has been shown that psychological factors also play a significant role in patients with organic dizziness and vertigo, e.g., 42.5% of patients with organic diagnoses suffer from a psychiatric comorbidity (Lahmann et al., 2015). Thus, considerable scores of anxiety and depression (VSS-A, HADS anxiety, and HADS depression) are found in our patients with non-organic diagnoses but in patients with organic diagnoses as well (see Figure 5).

This stresses the demand to perform multidimensional and multimodal diagnostic workup and therapy in patients with chronic dizziness and vertigo. Comprehensive early diagnosis of dizziness is very important to prevent further chronification and enable adequate treatment (Dieterich and Staab, 2017). Multimodal, systematically applied treatment plans have the potential to reduce morbidity and may induce persistent improvement (Dieterich and Staab, 2017).

## Conclusion

The study revealed that older patients with vertigo/dizziness in our vestibular rehabilitation program show characteristic features in comparison to younger patients. In the elderly mainly somatic deficits prevail while psychological factors such as anxiety play a minor role. In the future we will develop age related vestibular rehabilitation programs with a focus on physio- and occupational therapeutic interventions and less cognitive behavioral therapy for the elderly.

## Author Contributions

MD and HA substantial contributions to the conception and design of the work, the acquisition, analysis and interpretation of data for the work, drafting the work, and revising it critically for important intellectual content, and wrote the manuscript. SF, PK, and MZ substantial contributions to the acquisition and interpretation of data for the work, revising the work critically for important intellectual content. CK, OG-L, and OW substantial contributions to the conception and design of the work, the analysis and interpretation of data for the work, revising the work critically for important intellectual content. All authors gave their final approval of the version to be published and agree to be accountable for all aspects of the work in ensuring that questions related to the accuracy or integrity of any part of the work are appropriately investigated and resolved.

## Conflict of Interest Statement

The authors declare that the research was conducted in the absence of any commercial or financial relationships that could be construed as a potential conflict of interest.
